# Neonatal Suppurative Parotitis: A Case Report

**Published:** 2014-08-10

**Authors:** Mehdi Moradi

**Affiliations:** Hormozgan University of Medical Sciencesm, Hormozgan, Iran

**Keywords:** Neonatal Suppurative Parotitis, Newborn, Staphylococcus Aureus

**Dear Editor**

Neonatal suppurative parotitis (NSP) is rare; fewer than 90 cases were reported before 1970^[^^1^^]^. A study by Spiegel et al identified only 32 cases in the English literature between 1970 and 2004^[^^2^^]^. Another study by Ismail et al identified 12 new cases in the English literature since 2004^[^^3^^]^. We are reporting another case. Most cases were male (77%) and one third of them were preterm infants^[^^2^^,^^3^^]^.* Staphylococcus aureus* was the most frequent offending organism. Ascending infection from the oral cavity through Stensen’s duct or, less commonly, hematogenous seeding of the parotid gland have both been implicated in the development of NSP^[^^4^^]^. Several risk factors for the development of NSP are recognized: dehydration, prematurity, excessive oropharyngeal suction, prolonged gavaging, ductal obstruction and immune suppression^[^^2^^,^^3^^]^. Typically, infants present in the second week of life with parotid gland swelling, fever and erythema in the overlying skin. Purulent material can drain spontaneously or be expressed from the Stensen’s duct. 

**Fig. 1:**
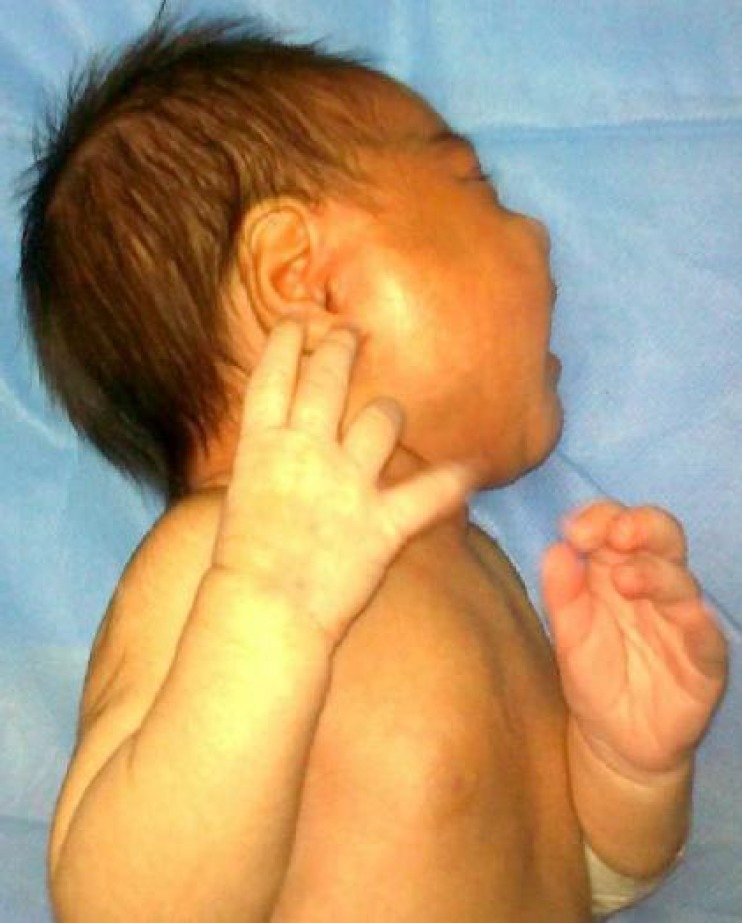
19-day-old female infant with right-sided periauricular swelling

 A 19-day-old female infant presented with a 3-day history of fever, irritability, and right-sided periauricular swelling [Fig. 1]. She was born at 37 weeks + 3days gestation via normal vaginal delivery. Pregnancy was uneventful. Birth weight was 3200 g. On admission the baby was irritable. Her weight was 3450 g, and rectal temperature 38°C. A diffuse, tender, unilateral swelling in the right parotid region was observed. It was warm to touch and fluctuated. The rest of the physical examination was unremarkable. The mother reported no history suggestive of mastitis or recent infection. 

Initial laboratory tests were as follows: white blood cell count 18600/mm^3^ with 53.4% neutrophils and 41% lymphocytes, hemoglobin 9.5 g/dl and erythrocyte sedimentation rate (ESR) 42 mm/h. Ultrasound examination of the mass demonstrated a cystic mass with septation and debris in the right parotid. Percutaneous aspiration of fluctuant area of the parotid gland was done and sample sent for culture. After obtaining blood and cerebrospinal fluid for culture empiric therapy with amikacin, vancomycin and ceftazidim was initiated. Surgical drainage was done 12 hours later.

 After four days of therapy, the swelling was not regressed. Culture of parotid abscess showed* Staphylococcus aureus* growth. With regard to antibiogram, clindamycin and meropenem was started and vancomycin and ceftazidim discontinued. Thereon blood culture, cerebrospinal fluid analysis and culture were normal. Daily drainage was not needed and after 10 days of therapy she was discharged.

 To our knowledge, acute bacterial infection of parotid gland in the neonatal age has not been reported yet from Iran. Our patient illustrates the typical manifestation of an acute suppurative parotitis.

 Our patient was female unlike the most of reported patients^[^^2^^,^^3^^]^. She had unilateral parotid gland involvement, like the most of cases^[^^2^^,^^3^^]^. One third of the patients were prematures^[^^2^^,^^3^^]^. With regard to the average worldwide prematurity rate of 9.6%^[^^5^^]^, it should be considered as a major risk factor for the infection. The patient was febrile on admission, however fever was reported in fewer than half of the patients^[^^3^^]^. As in our case, most patients had peripheral WBC count more than 15000/mm3^[^^2^^,^^3^^]^. Her ESR was 42mm/h which was elevated in only 20% of the patients^[^^2^^]^. *S. aureus *was the most frequently isolated pathogen in cultures of pus ^[^^2^^,^^3^^]^ same as in our case. However other Gram-positive organisms, Gram-negative organisms and anaerobic species were also isolated^[^^2^^,^^6^^]^. In our case blood culture was negative, like the most of cases^[^^2^^,^^3^^]^. She was product of a normal vaginal delivery. The causative agents are thought to be derived from the patients’ mouth flora. The newborns acquire their first microflora of the mouth, ear and skin from the mother’s birth canal during normal vaginal delivery^[^^7^^]^. She was breastfed, like the most of the patients with NSP^[^^2^^]^ and raises the possibility of insufficient breast-feeding as a responsible factor for dehydration in these patients. Ultrasound is a useful device for diagnosis, and excludes other predisposing factors like Stenson’s duct abnormality, sialolith, and parotid gland neoplasm. The shortest effective duration reported in treating NSP due to *S. aureus *and in the absence of septicemia was 7 days^[^^6^^]^. The prognosis seems to be excellent, however as complications salivary fistula, facial palsy, mediastinitis, septicemia and meningitis were reported^[^^1^^]^. No deaths were reported in the patients studied after 1970^[^^1^^]^. 
